# Considering serum alanine aminotransferase and gamma-glutamyltransferase levels together strengthen the prediction of impaired fasting glucose risk: a cross-sectional and longitudinal study

**DOI:** 10.1038/s41598-021-82981-z

**Published:** 2021-02-09

**Authors:** Ji Hye Jeong, Susie Jung, Kyu-Nam Kim

**Affiliations:** Department of Family Practice and Community Health, Ajou University School of Medicine, 164 Worldcup-ro, Yeongtong-gu, Suwon, 16499 Korea

**Keywords:** Biochemistry, Medical research

## Abstract

Emerging data suggest that an increase in serum alanine aminotransferase (ALT) and gamma-glutamyltransferase (GGT) as biomarkers of oxidative stress are associated with increased risk of impaired fasting glucose (IFG). The present study was an investigation of whether an increase in serum ALT and GGT had a combined effect on increasing IFG risk through cross-sectional and longitudinal studies. In the cross-sectional study, data were analyzed from 9937 subjects without diabetes who underwent health check-ups between 1999 and 2001 (baseline data). In the longitudinal study, 6390 subjects were analyzed who had been rechecked between 2009 and 2014, excluding IFG patients from baseline data. In cross-sectional analysis, adjusted odds ratio (OR) of IFG in the fourth quartile of both ALT and GGT was 1.829 (95% confidence interval [CI] 1.545–2.164) compared with the reference group (1st and 2nd quartiles of ALT and GGT). In longitudinal analysis, IFG probability increased gradually with an increase in the circulating levels of ALT and GGT. Adjusted hazard ratios for developing IFG in the fourth quartile of both ALT and GGT was 1.625 (95% CI 1.263–2.091) compared with the reference group (1st and 2nd quartiles). Increased serum ALT and GGT levels are well associated with IFG after potential confounders are adjusted for, and elevated ALT and GGT at the same time can have a combined effect in predicting the development of IFG.

## Introduction

According to an International Diabetes Federation report, the number of diabetic patients is expected to reach 578 million by 2030^1^, and in Korea, the less-than-1% diabetes prevalence of the 1970s increased to about 13.7% (4.8 million) of the population in 2014^2^. The fact that should be of interest is that impaired fasting glucose (IFG) is one of the high-risk categories for future diabetes, so about 8 millions of patients with IFG in Korea will necessarily further increase the diabetes prevalence in the future^2^. Therefore, it is important to early on identify and manage factors that contribute to IFG in Korea. In addition, recent studies have reported that microvascular and macrovascular damage in the cardiovascular system begins with IFG, a pre-diabetes condition^3,4^. Ultimately, identifying factors that contribute to the development of IFG can help predict the likelihood of progression to diabetes.

Emerging data refer to elevated levels of alanine aminotransferase (ALT) and gamma-glutamyltransferase (GGT) in the serum as predictive factors in the development of diabetes^5–8^. Serum ALT and GGT are enzymes that are widely used as biologic markers for liver damage caused by alcohol intake as well as for general liver function. In addition, elevated serum ALT is associtaed with increased the plasma levels of lipid peroxides, an oxidative stress marker^9^. GGT plays key roles in the extracellular metabolism of the antioxidant glutathione which leads to increased reactive oxygen species^10^. Thus, elevated ALT and GGT are potential markers for oxidative stress^11,12^, and cross-sectional and prospective studies have confirmed that they also contribute to cardiovascular disease and diabetes^5,13–15^. Furthermore, in the National Health and Nutrition Survey, which is recognized as a representative sample of the United States population, elevated ALT and GGT were associated with IFG as well as diabetes^16^. In a recent longitudinal study, our researchers found that ALT and GGT in the serum were independent risk factors for the onset of diabetes^17^. In addition, serum ALT and GGT in the fourth quartile compared with in the first and second quartiles showed combined effects on the development of diabetes. However, to our best knowledge, there has been no study on the combined effect of effects ALT and GGT in the development of IFG, a pre-diabetes stage. Therefore, we hypothesized that elevated levels of ALT and GGT in the serum were associated with the development of IFG, and we further investigated whether elevated levels of ALT and GGT in the blood combined to affect IFG development through cross-sectional and longitudinal studies.

## Methods

### Study population

We designed this study to incorporate cross-sectional and longitudinal analysis. First, the cross-sectional study consisted of subjects who had received checkups at the Health Promotion Center, Ajou University Hospital, Suwon, Korea, between 1999 and 2001(analysis for the first period). Second, we analyzed with a longitudinal study data from the above subjects who had undergone health rechecks from 2009 to 2014. If a person received more than two health checkups from 1999 to 2001, we used the first checkup results, and for anyone who received more than one recheck from 2009 to 2014, we used the results from the last checkup.

For the purposes of the cross-sectional study, we excluded patients who had received health examinations from 1999 to 2001 if they met any of the following conditions (n = 30,713): subjects diagnosed with diabetes; subjects with missing fasting blood glucose, serum ALT or serum GGT values; subjects with a history of chronic liver disease, such as hepatitis B or C or liver cirrhosis, or who were taking drugs that influenced liver function; subjects diagnosed with cardiovascular disease or cancer; subjects who had consumed enough alcohol to damage the liver (> 20 g/day in females or > 30 g/day in male)^18,19^. We further excluded subjects (n = 221) with more than three times (≥ 198U/L) the normal level of GGT because high levels can be caused by viruses or toxic substances. Therefore, we ultimately analyzed 9937 subjects. For the longitudinal study, we analyzed data from a final total of 6390 subjects after we excluded IFG patients from the data of the cross-sectional analysis among subjects who had been rechecked between 2009 and 2014, and used their first period data as baseline.

### Measurements

Blood was collected on the morning of the hospital visit after more than 8 h of fasting before the visit. Blood test items were fasting glucose, liver level, uric acid, total cholesterol, triglycerides (TG), high-density lipoprotein cholesterol (HDL-C), and low-density lipoprotein cholesterol (LDL-C). Then there was a break in testing for 5 min to measure blood pressure, which was measured twice with a mercury sphygmomanometer; we used the average of the two values. We defined hypertension as systolic blood pressure ≥ 140 mmHg or diastolic blood pressure ≥ 90 mm Hg or being on antihypertensive medications. We defined hyperlipidemia as LDL-C ≥ 160 mg/dL or TG ≥ 200 mg/dL or by the use of antihyperlipidemic medications. We defined IFG as fasting serum glucose of 100–125 mg/dL and diabetes as fasting serum glucose ≥ 126 mg/dL, or using oral hypoglycemic agents or insulin. We calculated body mass index (BMI) as weight (kilograms) divided by height squared (square meters). We also used a self-reporting questionnaire for history of hypertension, diabetes mellitus, hyperlipidemia, smoking status, and alcohol consumption, classifying smoking status as either a current smoker or a nonsmoker. We calculated individual daily alcohol consumption and then converted that to weekly consumption (grams of ethanol per week) using graduated frequency^18^. Medical history and use of medications were based on information obtained by interviewers trained in collecting data.

### Statistical analyses

We used descriptive analysis for general and baseline characteristics in cross-sectional and longitudinal analysis. In each cross-sectional study and longitudinal study, subjects were divided into quartiles according to serum ALT and GGT levels, and then we performed analysis of variance and trend analysis using polynomial contrasts. The distribution of alcohol consumption was right-skewed; therefore, we applied a natural-log transformation. In the cross-sectional study, we used multivariate logistic regression to analyze the IFG relationship according to serum ALT and GGT levels, which we also used to evaluate the combined effects of the two on IFG. In the longitudinal study analysis, we used Cox regression to analyze IFG development according to serum ALT and GGT at baseline, and also to evaluate the combined effects of the two on development of IFG. We included in the Cox regression analysis the covariates age, gender, BMI, log-transformed alcohol consumption, smoking status, hypertension, hyperlipidemia, and uric acid. We ran all statistical analyses using SPSS v20.0 software (SPSS Inc, Chicago, IL USA) and considered P < 0.05 statistically significant.

### Ethics approval

All methods were performed in accordance with the relevant guidelines and regulations. Written informed consent was obtained from subjects for permission to use their data. The institutional review board of Ajou University Hospital (Suwon, Republic of Korea) approved the study (Approval No.: AJIRB-MED-MDB-16-063).

## Results

**Figure 1:**
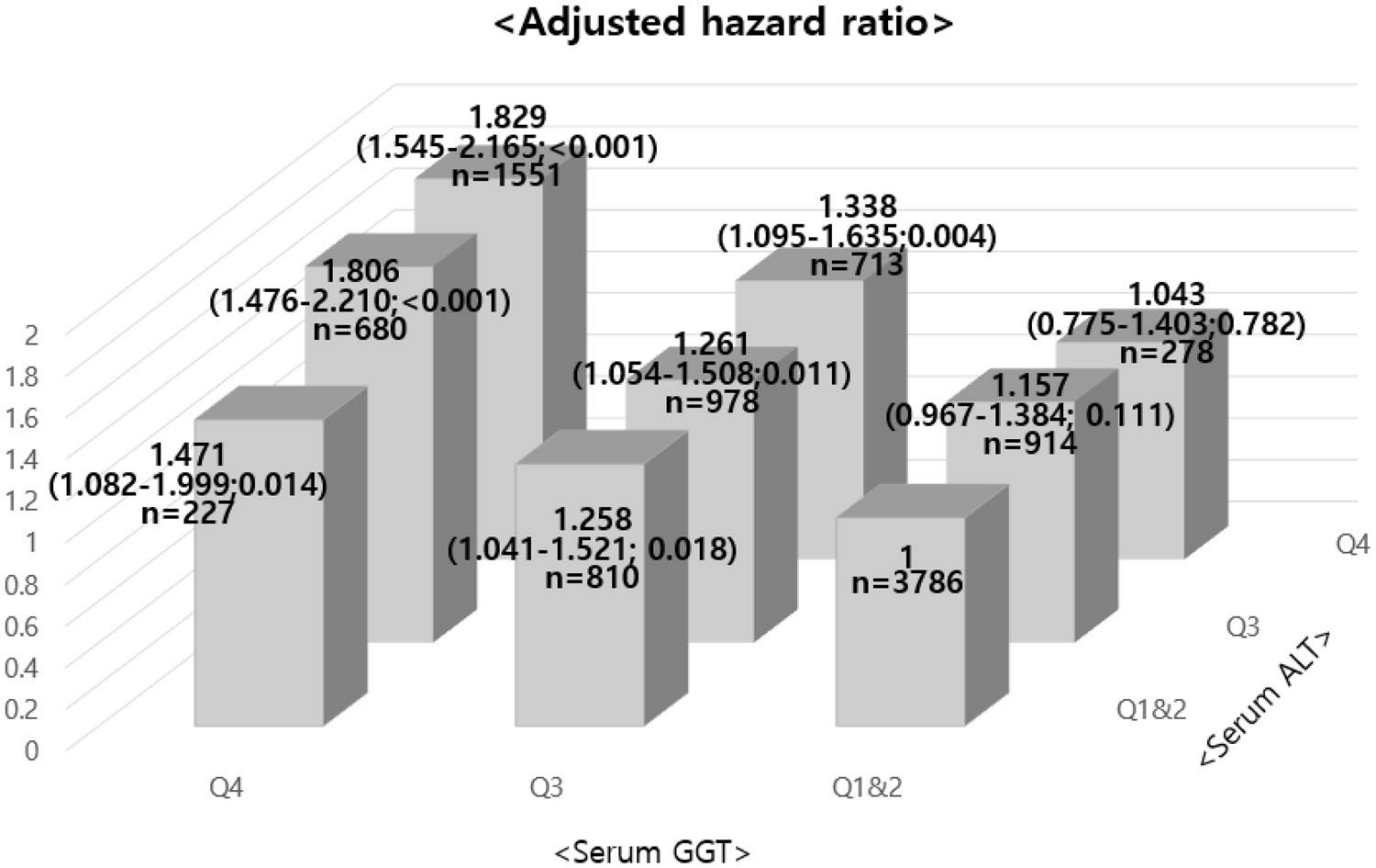
Adjusted hazard ratios ∗ (95% confidence interval; *P*) for the development of impaired fasting glucose according to the combined categories of ALT and GGT levels (1st and 2nd quartiles, 3rd quartile, and 4th quartile) in cross-sectional analysis. ∗ The model was adjusted for age, gender, body mass index, log-transformed weekly alcohol consumption, current smoker, hypertension, hyperlipidemia, and uric acid. *Q1* 1st quartile, *Q2* 2nd quartile, *Q3* 3rd quartile, *Q4* 4th quartile, *ALT* alanine aminotransferase, *GGT* gamma-glutamyltransferase.

Table 1 shows the general characteristics of the study subjects according to the quartiles of ALT and GGT in cross-sectional analysis. The ranges of the first to fourth quartiles of serum ALT were 3 to 17, 18 to 23, 24 to 34, and 35 to 201 IU/L, respectively, and the same ranges for the first to fourth quartiles of serum GGT were 4 to 13, 14 to 20, 21 to 35, and 36 to 198 IU/L, also respectively. The prevalence of hypertension, hyperlipidemia, smokers, and the alcohol consumption per week, BMI, and uric acid increased as the ALT quartile increased, and we observed the same trend with increasing GGT quartile. We also divided the study subjects into nine groups according to the ALT and GGT levels (1st and 2nd quartile, 3rd quartile, 4th quartile). Figure 1 shows the multivariate logistic analysis of IFG relationships according to serum ALT and GGT levels in the nine groups. When we considered ALT and GGT together, the odds ratio (OR) of IFG increased: Adjusted OR of IFG in the fourth quartiles of both ALT and GGT was 1.829 (95% confidence interval [CI] 1.545–2.164) compared with the reference group (1st and 2nd quartiles of ALT and GGT). This finding was supported by the fact that the interaction term for ALT and GGT was statistically significant (*P* < 0.001).

**Table 1:** The general characteristics of the study subjects by serum alanine aminotransferase and gamma-glutamyltransferase grading in cross-sectional analysis.

Characteristics	ALT grading, IU/L				
	Q1 (3–17)	Q2 (18–23)	Q3 (24–34)	Q4 (35–201)	*P* for trend
	n = 2607	n = 2216	n = 2572	n = 2542	
Age (years)	53.19 ± 8.17	56.24 ± 9.40	57.19 ± 9.14	55.70 ± 8.11	< 0.001
Men, no. (%)	614 (23.6)	1091 (49.2)	1849 (71.9)	2185 (86)	< 0.001
BMI (kg/m^2^)	21.82 ± 2.54	22.76 ± 2.77	23.69 ± 2.70	24.98 ± 2.78	< 0.001
SBP (mm Hg)	110.91 ± 14.61	116.38 ± 16.16	120.35 ± 15.99	123.80 ± 15.92	< 0.001
DBP (mm Hg)	69.58 ± 9.79	72.46 ± 10.53	74.52 ± 10.67	76.90 ± 11.05	< 0.001
FBG (mg/dL)	92.71 ± 7.90	94.92 ± 8.60	96.48 ± 9.04	98.36 ± 9.34	< 0.001
Total cholesterol (mg/dL)	174.48 ± 31.37	182.87 ± 32.15	190.17 ± 32.89	198.4 ± 34.99	< 0.001
HDL-C (mg/dL)	55.39 ± 12.53	53.13 ± 12.26	50.17 ± 12.30	47.62 ± 11.01	< 0.001
TG (mg/dL)	89.22 ± 46.72	109.56 ± 67.70	134.77 ± 85.90	173.12 ± 108.85	< 0.001
LDL-C (mg/dL)	103.10 ± 28.19	109.15 ± 28.81	113.85 ± 30.15	117.14 ± 33.28	< 0.001
Alcohol (g/wk)	19.96 ± 61.57	36.43 ± 79.32	59.06 ± 100.52	75.94 ± 123.73	< 0.001
Uric acid (mg/dL)	4.29 ± 1.12	4.86 ± 3.13	5.40 ± 1.32	5.99 ± 1.38	< 0.001
Current smoker, no. (%)	362 (13.9)	569 (25.7)	977 (38.0)	1206 (47.4)	< 0.001
HTN, no. (%)	81 (3.2)	134 (6.2)	208 (8.3)	238 (9.7)	< 0.001
Hyperlipidemia, no. (%)	43 (1.7)	81 (3.8)	141 (5.7)	201 (8.2)	< 0.001

	GGT grading, IU/L				
	Q1 (4–13)	Q2 (14–20)	Q3 (21–35)	Q4 (36–198)	*P* for trend
	n = 2479	n = 2499	n = 2501	n = 2458	
Age (years)	53.12 ± 8.41	55.81 ± 9.21	56.61 ± 8.94	56.65 ± 8.24	< 0.001
Men, no. (%)	284 (11.5)	1202 (48.1)	1998 (79.9)	2255 (91.7)	< 0.001
BMI (kg/m^2^)	21.89 ± 2.52	22.80 ± 2.83	23.72 ± 2.82	24.87 ± 2.74	< 0.001
SBP (mm Hg)	110.82 ± 15.06	115.83 ± 15.83	120.19 ± 15.73	124.69 ± 15.68	< 0.001
DBP (mm Hg)	69.52 ± 9.57	71.87 ± 10.20	74.07 ± 10.71	78.08 ± 11.08	< 0.001
FBG (mg/dL)	92.28 ± 7.77	94.57 ± 8.35	96.41 ± 8.88	99.27 ± 9.39	< 0.001
Total cholesterol (mg/dL)	172.95 ± 29.54	182.92 ± 33.23	190.57 ± 32.84	199.79 ± 34.75	< 0.001
HDL-C (mg/dL)	56.61 ± 12.21	52.92 ± 12.42	49.07 ± 11.88	48.21 ± 11.16	< 0.001
TG (mg/dL)	83.06 ± 40.68	104.16 ± 56.36	137.35 ± 79.03	184.04 ± 115.66	< 0.001
LDL-C (mg/dL)	101.50 ± 26.36	111.02 ± 29.72	115.21 ± 30.50	115.65 ± 34.44	< 0.001
Alcohol (g/wk)	8.20 ± 33.46	26.56 ± 72.58	51.90 ± 89.11	106.26 ± 133.96	< 0.001
Uric acid (mg/dL)	4.04 ± 1.00	4.81 ± 1.20	5.61 ± 2.98	6.10 ± 1.30	< 0.001
Current smoker, no. (%)	140 (5.6)	586 (23.4)	1040 (41.6)	1348 (54.8)	< 0.001
HTN, no. (%)	73 (3.0)	144 (5.9)	196 (8.1)	248 (10.4)	< 0.001
Hyperlipidemia, no. (%)	31 (1.3)	87 (3.6)	128 (5.3)	220 (9.3)	< 0.001

*Q1* 1st quartile, *Q2* 2nd quartile, *Q3* 3rd quartile, *Q4* 4th quartile, *ALT* alanine aminotransferase, *BMI* body mass index, *SBP* systolic blood pressure, *DBP* diastolic blood pressure, *FBG* fasting blood glucose, *HDL-C* high-density lipoprotein cholesterol, *TG* triglycerides, *LDL-C* lowdensity lipoprotein cholesterol, *HTN* hypertension, *GGT* gamma-glutamyltransferase.

Table 2 shows baseline characteristics according to the quartiles of ALT and GGT in longitudinal analysis. As with the cross-sectional study analysis, the prevalence and number of risk factors of IFG increased as the ALT and GGT quartiles increased. The mean follow-up periods from the first to fourth quartiles of serum ALT were 9.86, 9.34, 9.35, and 9.42 years, respectively, and serum ALT ranges from the first to the fourth quartile were 3–16, 17–21, 22–31, and 32–198 IU/L, also respectively. The mean follow-up periods from the first to fourth quartiles of serum GGT were 9.50, 9.46, 9.54, and 9.51 years, respectively, and serum GGT ranges from the first to the fourth quartile were 4–12, 13–17, 18–29, and 30–198 IU/L, also respectively.

**Table 2:** The baseline characteristics of the study subjects by serum alanine aminotransferase and gamma-glutamyltransferase grading in longitudinal analysis.

Characteristics	ALT grading, IU/L				
	Q1 (3–16)	Q2 (17–21)	Q3 (22–31)	Q4 (32–198)	*P* for trend
	n = 1724	n = 1400	n = 1719	n = 1547	
Age (years)	52.19 ± 7.58	54.90 ± 8.96	56.03 ± 9.04	54.92 ± 8.18	< 0.001
Men, no. (%)	348 (20.2)	563 (40.2)	1115 (64.9)	1288 (83.3)	< 0.001
BMI (kg/m^2^)	21.52 ± 2.46	22.30 ± 2.69	23.01 ± 2.62	24.37 ± 2.70	< 0.001
SBP (mm Hg)	108.9 ± 13.74	113.61 ± 14.92	117.54 ± 16.06	120.90 ± 15.29	< 0.001
DBP (mm Hg)	68.61 ± 9.49	70.96 ± 9.99	73.09 ± 10.52	75.03 ± 10.61	< 0.001
FBG (mg/dL)	89.95 ± 5.59	90.77 ± 5.32	91.25 ± 5.54	91.94 ± 5.14	< 0.001
Total cholesterol (mg/dL)	171.03 ± 30.40	179.39 ± 30.21	186.00 ± 31.73	193.47 ± 33.91	< 0.001
HDL-C (mg/dL)	55.67 ± 12.45	54.37 ± 12.56	51.83 ± 12.81	48.38 ± 11.38	< 0.001
TG (mg/dL)	84.61 ± 41.70	99.77 ± 63.10	121.08 ± 73.01	157.90 ± 104.22	< 0.001
LDL-C (mg/dL)	100.36 ± 27.76	106.42 ± 27.74	111.25 ± 29.09	115.10 ± 31.97	< 0.001
Alcohol (g/wk)	17.07 ± 52.40	31.11 ± 77.88	47.31 ± 90.14	67.70 ± 119.01	< 0.001
Uric acid (mg/dL)	4.19 ± 1.07	4.59 ± 1.20	5.17 ± 1.30	5.86 ± 1.38	< 0.001
Current smoker, no. (%)	224 (13.0)	328 (23.4)	627 (36.5)	716 (46.3)	< 0.001
HTN, no. (%)	39 (2.3)	52 (3.8)	95 (5.7)	115 (7.7)	< 0.001
Hyperlipidemia, no. (%)	19 (1.1)	38 (2.8)	74 (4.4)	94 (6.3)	< 0.001
Internal years	9.86 ± 3.48	9.34 ± 3.51	9.35 ± 3.53	9.42 ± 3.54	

	GGT grading, IU/L				
	Q1 (4–12)	Q2 (13–17)	Q3 (18–29)	Q4 (30–198)	*P* for trend
	n = 1581	n = 1497	n = 1736	n = 1576	
Age (years)	52.32 ± 7.86	54.69 ± 2.27	55.26 ± 8.65	55.58 ± 8.07	< 0.001
Men, no. (%)	134 (8.5)	518 (34.6)	1252 (72.1)	1410 (89.5)	< 0.001
BMI (kg/m^2^)	21.66 ± 2.42	22.20 ± 2.64	23.06 ± 2.80	24.25 ± 2.69	< 0.001
SBP (mm Hg)	109.13 ± 14.41	112.64 ± 15.01	116.59 ± 14.99	122.10 ± 15.31	< 0.001
DBP (mm Hg)	68.83 ± 9.41	70.56 ± 9.90	71.87 ± 10.28	76.22 ± 10.68	< 0.001
FBG (mg/dL)	89.79 ± 5.62	90.57 ± 5.43	91.31 ± 5.46	92.13 ± 5.04	< 0.001
Total cholesterol (mg/dL)	169.97 ± 28.84	178.89 ± 31.65	185.19 ± 31.52	194.81 ± 33.51	< 0.001
HDL-C (mg/dL)	56.85 ± 12.27	54.83 ± 12.81	50.40 ± 12.13	48.65 ± 11.63	< 0.001
TG (mg/dL)	78.96 ± 36.65	93.94 ± 48.03	121.84 ± 71.99	165.58 ± 106.76	< 0.001
LDL-C (mg/dL)	98.91 ± 25.48	107.36 ± 28.92	111.50 ± 29.30	114.72 ± 32.17	< 0.001
Alcohol (g/wk)	7.19 ± 30.75	18.35 ± 51.64	43.51 ± 93.78	91.80 ± 123.37	< 0.001
Uric acid (mg/dL)	3.94 ± 0.92	4.53 ± 1.15	5.27 ± 1.23	5.98 ± 1.31	< 0.001
Current smoker, no. (%)	75 (4.7)	259 (17.3)	713 (41.1)	848 (53.8)	< 0.001
HTN, no. (%)	39 (2.5)	51 (3.5)	95 (5.6)	116 (7.6)	< 0.001
Hyperlipidemia, no. (%)	14 (0.9)	37 (2.6)	74 (4.4)	100 (6.6)	< 0.001
Follow-up period (years)	9.50 ± 3.50	9.46 ± 3.49	9.54 ± 3.50	9.51 ± 3.59	

*Q1* 1st quartile, *Q2* 2nd quartile, *Q3* 3rd quartile, *Q4* 4th quartile, *ALT* alanine aminotransferase, *BMI* body mass index, *SBP* systolic blood pressure, *DBP* diastolic blood pressure, *FBG* fasting blood glucose, *HDL-C* high-density lipoprotein cholesterol, *TG* triglycerides, *LDL-C* lowdensity lipoprotein cholesterol, *HTN* hypertension, *GGT* gamma-glutamyltransferase.

We performed the Cox proportional hazards regression analysis for the hazard ratios of IFG in the second to fourth quartiles of serum ALT compared with the lowest quartile after we adjusted for traditional and novel risk factors (age, gender, BMI, hypertension, hyperlipidemia, alcohol consumption per week, current smokers, and uric acid; Table 3). Compared with individuals in the first quartile of serum ALT, the adjusted hazard ratios (95% CI) of the second to fourth quartiles were 1.456 (1.167–1.817), 1.519 (1.224–1.884), and 1.557 (1.237–1.939), respectively; the hazard ratio of the highest quartile was attenuated after we adjusted confounding variables, but it remained statistically significant. For GGT as well, the adjusted hazard ratios (95% CI) of the second to highest quartiles were 1.373 (1.070–1.761), 1.728 (1.343–2.222), and 1.928 (1.466–2.535), respectively, compared with individuals in the lowest quartile. The hazard ratios of the third and highest quartiles of serum GGT were also attenuated after we adjusted for confounders but remained statistically significant.

**Table 3:** Hazard ratios for developing impaired fasting glucose by serum alanine aminotransferase and gamma-glutamyltransferase at baseline in longitudinal analysis.

	Serum ALT, IU/L (confidence interval; *P*)				
	Q1 (3–16 )	Q2 (17–21 )	Q3 (22–31)	Q4 (32–198)	*P* for trend
Model 1	1	1.814 (1.468–2.246;); < 0.001	2.190 (1.800–2.664); < 0.001	2.649 (2.186–3.209); < 0.001	< 0.001
Model 2	1	1.474 (1.186–1.832); < 0.001	1.510 (1.221–1.869); < 0.001	1.614 (1.289–2.021); < 0.001	< 0.001
Model 3	1	1.456 (1.167–1.817);0.001	1.519 (1.224–1.884); < 0.001	1.557 (1.237–1.959); < 0.001	< 0.001

	Serum GGT, IU/L (confidence interval; P)				
	Q1 (4–12)	Q2 (13–17)	Q3 (18–29)	Q4 (30–198)	
Model 1	1	1.539 (1.217–1.946); < 0.001	2.232 (1.803–2.763); < 0.001	3.064 (2.493–3.767); < 0.001	< 0.001
Model 2	1	1.331 (1.045–1.695);0.021	1.702 (1.332–2.174); < 0.001	2.010 (1.548–2.610); < 0.001	< 0.001
Model 3	1	1.373 (1.070–1.761);0.013	1.728 (1.343–2.222); < 0.001	1.928 (1.466–2.535); < 0.001	< 0.001

Model 1, unadjusted; Model 2, after adjustment for age, gender and body mass index;Model 3, model 2 plus after adjustment for log-transformed weekly alcohol consumption, current smoker, hypertension, hyperlipidemia, and uric acid.

*Q1* 1st quartile, *Q2* 2nd quartile, *Q3* 3rd quartile, *Q4* 4th quartile.

**Figure 2:**
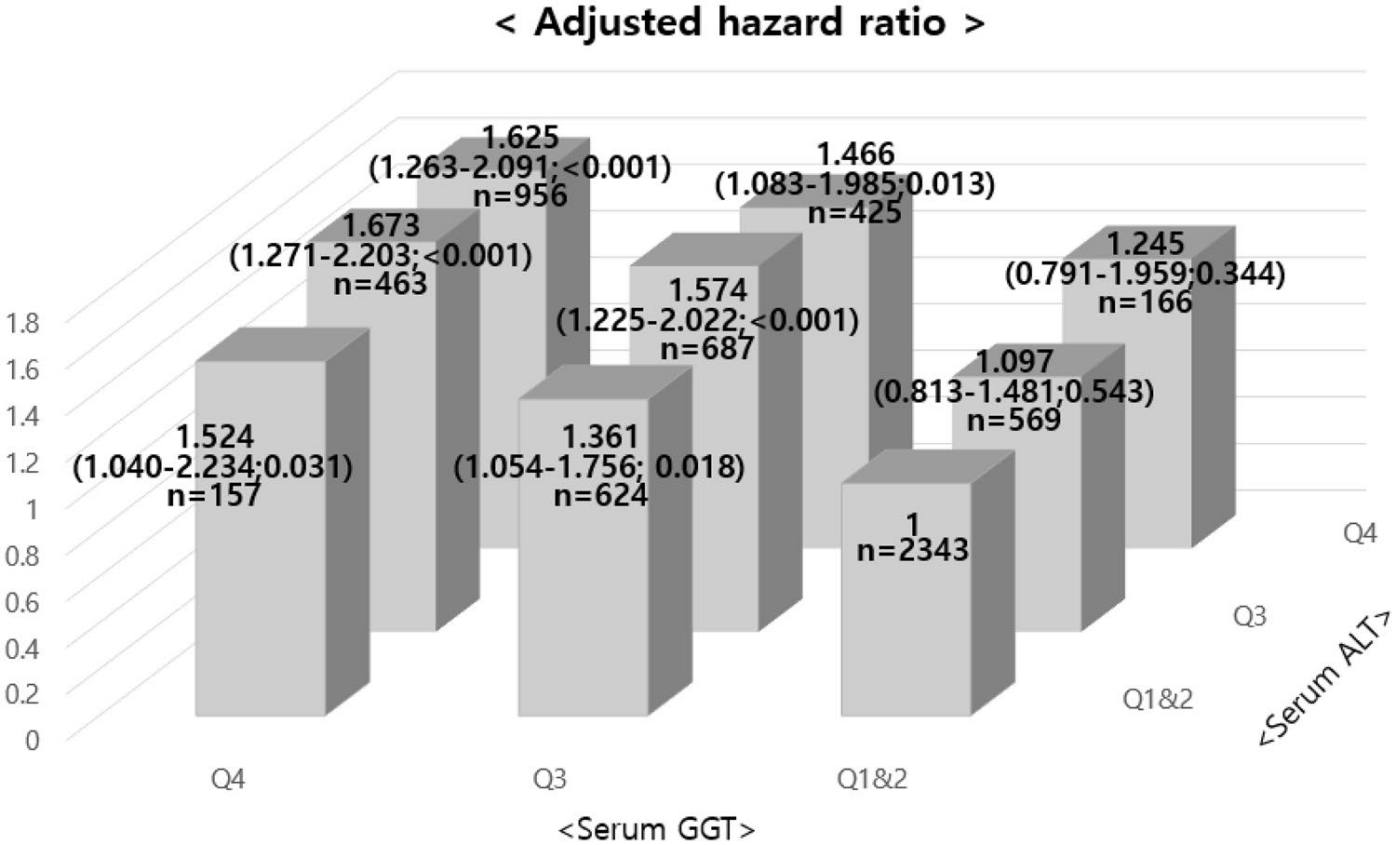
Adjusted hazard ratios ∗ (95% confidence interval; P) for the development of impaired fasting glucose according to the categories of combined ALT and GGT levels (1st and 2nd quartile, 3rd quartile, and 4th quartile) in longitudinal analysis. ∗ The model was adjusted for age, gender, body mass index, log-transformed weekly alcohol consumption, current smoker, hypertension, hyperlipidemia, and uric acid. *Q1* 1st quartile, *Q2* 2nd quartile, *Q3* 3rd quartile, *Q4* 4th quartile, *ALT* alanine aminotransferase, *GGT* gamma-glutamyltransferase.

We also divided the study subjects into nine groups (1st & 2nd quartile, 3rd quartile, 4th quartile) with the first and second quartiles of serum ALT and GGT levels as reference group in the longitudinal analysis (Fig. 2). We found that considering ALT and GGT together strengthened the hazards ratio relationship for the development of IFG. Adjusted hazard ratio for new onset IFG in the fourth quartiles of both ALT and GGT was 1.625 (95% CI 1.263–2.091) compared with the reference group. We analyzed the interaction term between ALT and GGT, and found that it was statistically significant (*P* < 0.001). Therefore, the above finding were supported by this fact.

## Discussion

With the present study, we investigated whether elevated levels of ALT and GGT in the serum were associated with IFG through cross-sectional and longitudinal analysis, and we found that IFG development did indeed significantly increase according to serum ALT and GGT quartiles after we adjusted for established diabetes risk factors (age, gender, BMI, log-transformed alcohol consumption, smoking status, hypertension, and hyperlipidemia), and novel risk factor (uric acid). In addition, serum ALT and GGT in the fourth quartile compared with in the first and second quartiles showed a synergistic effect on new onset IFG.

Our results are in accordance with studies of the relationship between ALT and IFG in adults and students. Researchers who investigated the possible correlation between levels of serum ALT enzymes and IFG in Western and Asian adults found that the cumulative incidences of IFG were significantly higher in the highest quartiles of ALT enzymes than in the lowest quartiles^16,20^. A cross-sectional study of 6,716 students also demonstrated that ALT levels were significantly and positively related to IFG risk^21^. Furthermore, in a longitudinal (7 years) study of electronics workers, the OR when ALT increased to borderline was 2.664 (95% CI 1.214–5.849), which was associated with incidence of IFG or diabetes^22^.

Cross-sectional and prospective studies of the relationship between serum GGT and IFG showed similar findings to ours. In a cross-sectional study of community-dwelling subjects, the OR (95% CI) for IFG compared with the subjects in the lowest quartile of serum GGT was 1.91 (1.31–2.78) for second quartiles, 2.41 (1.63–3.57) for third quartiles, and 3.24 (2.03–5.17) for highest quartiles after adjustment for multiple traditional and novel risk factors^23^. A longitudinal study that followed about 3000 persons for more than 7 years presented relative risks for IFG compared with serum GGT < 16 U/L of 1.23 (95% CI 0.79–1.90), 1.50 (CI 0.97–2.32), and 1.70 (CI 1.07–2.71) with serum GGT of 16–24, 25–43, and ≥ 44 U/L), respectively, after adjustment for potential risk factors^24^.

Although the exact mechanism of ALT and GGT in IFG development is not yet known, recent research suggests several hypotheses. ALT is a representative biomarker for liver function and also considered an epidemiologic marker for non-alcoholic fatty liver because it is related to insulin sensitivity^25^. Non-alcoholic fatty liver has been linked to dysglycemia in the body^26^, and elevated ALT may contribute to the development of IFG because it is linked to insulin resistance in the liver^27^. GGT is typically a biomarker for alcoholic liver disease, but in a recent study, it was utilized as an index for oxidative stress in connection with glutathione^28^. That is, GGT in the serum increases in response to oxidative stress, which then increases the oxidative stress in cell levels^29^. The pancreas is an organ that regulates insulin secretion, and has it fewer antioxidant enzymes than other body organs^30^. Therefore, an increase in GGT, which means an increase in oxidative stress, may be a factor in reducing insulin secretion by damaging the pancreatic beta cells. These mechanisms may explain whether ALT and GGT are involved in the development of IFG.

We investigated whether there was a synergism between serum ALT and GGT in the development of IFG, and we found that the multivariate adjusted OR for development of IFG in the fourth quartile of serum ALT and GGT was significantly higher than that for the reference group. Taken together, the simultaneous elevation of ALT and GGT as biomarkers of oxidative stress may contribute more to the development of IFG by damaging the pancreatic beta cells than the elevation of only one indicator. These findings are supported by the findings that increased levels of both ALT and GGT are associated with subclinical inflammation and oxidative stress^9,31,32^.

Our data showed that the upper levels of ALT and GGT in the third quartile were 34, 35, 31 and 29 IU/L in cross-sectional and longitudinal analyses; these values are considered within the normal range in most laboratories. As the quartiles of ALT and GGT increased, the OR for incident IGF also increased, a relationship that remained statistically significant. Our results are consistent with findings related to analyses of medical checkup data that were similar to our study design^26,33^. Overall, even in the normal range, high levels of ALT and GGT in the serum may indicate increased oxidative stress, and these results might decrease pancreatic function and contribute to the incidence of IFG.

The present study had several strengths and limitations. First, to the best of our knowledge, this was the first cross-sectional and longitudinal examination of the combined effects of serum ALT and GGT on the onset of IFG in a general population. Second, this study included a relatively large number of subjects and had a long follow-up period. Third, this study did not include ALT and GGT during the follow-up period. However, the results showing that serum ALT and GGT in cross-sectional and longitudinal studies are associated with IFG may mean that elevated ALT and GGT at baseline are maintained even at the end of the study. Fourth, our data are not representative of the entire population because we studied nonrandomly selected patients at just one center. Despite these potential limitations, our findings from a large cohort support the conclusion that elevated serum ALT and GGT are associated with a higher risk for developing IFG even levels are still normal.

In conclusion, the current study shows that the combined effect of serum ALT and GGT is associated with an increased risk of IFG, and the simultaneous elevation of both indicators seems to help predict future IFG or diabetes incidence. In addition, the greater association between the elevation of both serum ALT and GGT and IFG incidence suggests that even those who do not currently have IFG, physicians should carefully pay attention to these patients to prevent cardiovascular events.

## Data Availability

All data generated or analyzed during the current study are included in this article.
