# Author Correction: Considering serum alanine aminotransferase and gamma-glutamyltransferase levels together strengthen the prediction of impaired fasting glucose risk: a cross-sectional and longitudinal study

**DOI:** 10.1038/s41598-021-90021-z

**Published:** 2021-05-25

**Authors:** Ji Hye Jeong, Susie Jung, Kyu-Nam Kim

**Affiliations:** Department of Family Practice and Community Health, Ajou University School of Medicine, 164 Worldcup-ro, Yeongtong-gu, Suwon, 16499 Korea

Correction to: *Scientific Reports* 10.1038/s41598-021-82981-z, published online 09 February 2021

This Article contains errors, where the legends for Table 1 and Table 2 are incomplete.

Table 1 legend “The general characteristics of the study subjects by serum alanine aminotransferase and gammaglutamyltransferase grading in cross-sectional analysis. *Q1* 1st quartile, *Q2* 2nd quartile, *Q3* 3rd quartile, *Q4* 4th quartile, *ALT* alanine aminotransferase, *BMI* body mass index, *SBP* systolic blood pressure, *DBP* diastolic blood pressure, *FBG* fasting blood glucose, *HDL-C* high-density lipoprotein cholesterol, *TG* triglycerides, *LDLC* lowdensity lipoprotein cholesterol, *HTN* hypertension, *GGT* gamma-glutamyltransferase.”

should read:

“The general characteristics of the study subjects by serum alanine aminotransferase and gammaglutamyltransferase grading in cross-sectional analysis. Data are expressed as mean ± standard deviation or as number (percentage). *Q1* 1st quartile, *Q2* 2nd quartile, *Q3* 3rd quartile, *Q4* 4th quartile, *ALT* alanine aminotransferase, *BMI* body mass index, *SBP* systolic blood pressure, *DBP* diastolic blood pressure, *FBG* fasting blood glucose, *HDL-C* high-density lipoprotein cholesterol, *TG* triglycerides, *LDLC* lowdensity lipoprotein cholesterol, *HTN* hypertension, *GGT* gamma-glutamyltransferase.”

Table 2 legend “The baseline characteristics of the study subjects by serum alanine aminotransferase and gammaglutamyltransferase grading in longitudinal analysis. *Q1* 1st quartile, *Q2* 2nd quartile, *Q3* 3rd quartile, *Q4* 4th quartile, *ALT* alanine aminotransferase, *BMI* body mass index, *SBP* systolic blood pressure, *DBP* diastolic blood pressure, *FBG* fasting blood glucose, *HDL-C* high-density lipoprotein cholesterol, *TG* triglycerides, *LDLC* lowdensity lipoprotein cholesterol, *HTN* hypertension, *GGT* gamma-glutamyltransferase.”

should read:

“The baseline characteristics of the study subjects by serum alanine aminotransferase and gammaglutamyltransferase grading in longitudinal analysis. Data are expressed as mean ± standard deviation or as number (percentage). *Q1* 1st quartile, *Q2* 2nd quartile, *Q3* 3rd quartile, *Q4* 4th quartile, *ALT* alanine aminotransferase, *BMI* body mass index, *SBP* systolic blood pressure, *DBP* diastolic blood pressure, *FBG* fasting blood glucose, *HDL-C* high-density lipoprotein cholesterol, *TG* triglycerides, *LDLC* lowdensity lipoprotein cholesterol, *HTN* hypertension, *GGT* gamma-glutamyltransferase.”

In addition, in Table 1, Characteristics,

“Internal years”

should read:

“Follow up period (years)”.

